# Artificial intelligence agents in healthcare research: A scoping review

**DOI:** 10.1371/journal.pone.0342182

**Published:** 2026-02-10

**Authors:** Basile Njei, Yazan A. Al-Ajlouni, Ulrick Sidney Kanmounye, Sarpong Boateng, Guy Loic Nguefang, Nelvis Njei, Shadi Hamouri, Ahmad F. Al-Ajlouni

**Affiliations:** 1 Section of Digestive Diseases, Department of Medicine, Yale University, New Haven, Connecticut, United States of America; 2 Engelhardt School of Global Health and Bioethics, Euclid University, Bangui, Central African Republic; 3 Artificial Intelligence Programme, University of Cumbria, Carlisle, United Kingdom; 4 Ohio University Heritage College of Osteopathic Medicine, Athens, Ohio, United States of America; 5 Yale Liver Center, Yale New Haven Health, New Haven, Connecticut, United States of America; 6 International Medicine Program, Yale Medicine, New Haven, Connecticut, United States of America; 7 Montefiore Medical Center/Einstein School of Medicine, Bronx, New York, United States of America; 8 Research Department, Association of Future African Neurosurgeons, Yaounde, Cameroon; 9 Yale Affiliated Hospitals Program, Bridgeport, Connecticut, United States of America; 10 Texas Tech University Health Science Center, Odessa, Texas, United States of America; 11 Machine Learning and Artificial Intelligence, Ellicott City, Maryland, United States of America; 12 Al-Balqa Applied University, Salt, Jordan; 13 Jordan University of Science and Technology, Irbid, Jordan; Cardiff Metropolitan University, UNITED KINGDOM OF GREAT BRITAIN AND NORTHERN IRELAND

## Abstract

**Introduction:**

Artificial Intelligence (AI) agents are rapidly transforming healthcare delivery, enabling real-time decision support and sophisticated patient interaction at scale. However, the scientific landscape of this rapidly growing, multidisciplinary field remains fragmented, with technical innovation outpacing translational research and the establishment of ethical governance frameworks. To address this gap, we conducted a comprehensive scoping review analysis of AI agent research in healthcare.

**Methods:**

We followed scoping review methodology (PRISMA-ScR guidelines). Searches across PubMed, Web of Science, arXiv, and medRxiv were conducted from January 2015 to December 7, 2025.

**Results:**

The search identified 1,070 records, of which 43 studies were ultimately included after full-text review. Of these 43 included studies, 36 were published in 2025. Systems were categorized into 8 conversational agents, 17 workflow/automation assistants, and 18 multimodal decision support agents. The core mechanism across all archetypes was external tool use (e.g., retrieval-augmented generation or code execution) for grounding and iterative self-correction (e.g., multi-agent debate or self-debugging loops) for refinement. Evaluation settings were predominantly simulated environments or laboratory studies, with few clinical pilots or real-world deployments. Primary reported outcomes focused on process measures (efficiency) and diagnostic accuracy; clinical outcomes and safety endpoints were rarely addressed.

**Conclusion:**

Agentic AI systems are rapidly evolving from conceptual frameworks to functional prototypes, primarily targeting complex decision-making and workflow automation. While agentic capabilities are increasingly integrated, research heavily favors simulated evaluations. Future research must prioritize clinical trials and the robust assessment of safety, usability, and clinical efficacy before widespread adoption.

## Introduction

Artificial Intelligence (AI) refers to computational systems capable of performing tasks that typically require human intelligence, such as reasoning, learning, and decision-making [1]. These systems leverage algorithms and data to identify patterns, make predictions, and adapt to new information. Within this broad domain, AI agents represent a specialized class of intelligent entities distinguished by their semi-autonomy, context awareness, and adaptive learning capabilities [1]. Unlike static algorithms, AI agents are designed to interact dynamically with their environment, continuously process incoming data, and adjust their actions based on evolving clinical contexts. In healthcare, AI agents represent a distinct class of computational entities characterized by semi-autonomy, context awareness, and adaptive learning capabilities that enable them to interact dynamically with clinical environments and patient data [2]. Unlike conventional algorithms, these agents are designed to reason, learn, and make decisions in real time, thereby offering the potential to augment clinical judgment and streamline healthcare delivery at scale [3]. Their evolution has been accelerated by advances in natural language processing, reinforcement learning, and multimodal architectures, which collectively support more sophisticated conversational interfaces and decision‑support functionalities [4,5].

The integration of AI agents into healthcare systems has been driven by several converging factors. The COVID‑19 pandemic catalyzed the adoption of remote care modalities, creating an urgent need for digital tools capable of sustaining patient engagement and clinical continuity without physical contact [6]. Concurrently, the maturation of large language models (LLMs) and reinforcement learning frameworks has enabled the development of agents that can interpret complex clinical narratives, manage multimodal inputs, and deliver personalized recommendations [7]. These capabilities have positioned AI agents as pivotal instruments in domains such as telemedicine, clinical decision support, mental health interventions, and workflow automation [8,2].

Despite these advances, the literature reveals a persistent gap between technical innovation and real‑world implementation. While engineering‑centric studies emphasize architectural optimization and algorithmic performance, translational research addressing usability, safety, and clinical outcomes remains comparatively underrepresented [9]. In addition, ethical considerations, including transparency, accountability, and patient trust, have gained prominence only in recent years, underscoring the need for governance frameworks that can accompany technological progress [7,10]. This fragmentation has impeded the establishment of standardized deployment protocols and evaluation metrics, limiting the scalability and generalizability of AI agents across diverse healthcare settings.

The purpose of this scoping review is to systematically map the current evidence on agentic AI in healthcare, focusing specifically on systems that transcend simple prompt-response interactions by demonstrating autonomous planning, tool utilization, and self-correction. We aim to achieve three primary objectives: (1) to delineate the core characteristics and functional archetypes of agentic AI systems reported in the literature; (2) to summarize the technical mechanisms (e.g., memory, tool-use, multi-agent frameworks) used to confer agency within these clinical and research applications; and (3) to identify critical gaps in the evaluation of these systems, particularly regarding their long-term clinical efficacy, safety, and integration into existing real-world healthcare workflows.

## Methods

### Study design and methodological orientation

The scoping review followed the stages proposed by Arksey and O’Malley, namely identifying the research question, locating relevant evidence, study selection, data charting, and collating, summarizing, and reporting the results, and reporting [11]. Reporting was aligned with the Preferred Reporting Items for Systematic Reviews and Meta‑Analyses extension for Scoping Reviews (PRISMA-ScR) to promote transparent documentation of eligibility criteria, sources, selection procedures, data items, and synthesis methods [12].

### Protocol and registration

A protocol was drafted a priori by the authors to prespecify the research questions, eligibility criteria, information sources, draft search strategies, screening and data charting procedures, and planned analyses. The protocol was archived internally with a date‑stamped record before database searching. Given that prevailing registries (e.g., PROSPERO) do not routinely index scoping review protocols without a clinical outcome synthesis plan, no external registration was undertaken. Any deviations from the prespecified plan are reported in the relevant subsections below.

### Eligibility criteria

The review was structured using the Population–Concept–Context (PCC) framework. The population comprised studies describing artificial intelligence systems applied within healthcare or biomedical research workflows. The concept of interest was agentic AI, operationally defined as a system built around an LLM that autonomously plans, reasons, and acts toward a complex goal through a self-correcting loop. Such systems incorporate a planning module to decompose tasks, a memory layer to retain intermediate states, and an executor capable of invoking external tools or APIs beyond the intrinsic capabilities of the base LLM. This definition distinguishes agentic AI from conventional LLMs and chatbots, which generate single-turn or static responses without iterative planning, persistent memory, or tool integration. Systems limited to prompt engineering, retrieval-augmented generation without autonomous orchestration, or rule-based multi-agent architectures were excluded. The context was restricted to healthcare delivery, clinical decision support, biomedical research, or related domains; studies focused on non-health applications, agent-based simulations, or purely theoretical frameworks were excluded. These operational boundaries ensured that included studies represented goal-directed, LLM-centric agents with autonomy and adaptability rather than generic conversational models or traditional machine learning pipelines.

### Information sources

Four sources were queried to balance coverage of biomedicine, computer science, and rapidly disseminated preprints. These were PubMed, Web of Science Core Collection, arXiv, and medRxiv. arXiv and medRxiv were included to capture emergent work at the interface of computer science and medicine. The inclusion of pre-print servers (arXiv, medRxiv) was a deliberate methodological choice for this scoping review, necessitated by the unprecedented velocity and interdisciplinary nature of agentic AI research. This approach ensured that the mapping of the evidence base was current and complete, capturing emerging technical innovations typically disseminated first in computer science and preliminary clinical findings prior to formal peer review.

Searches were conducted from January 2015 onward. This start date was selected because the mid-2010s mark a widely recognized inflection point in applied artificial intelligence, characterized by the maturation of deep learning architectures, scalable training on large datasets, and the increasing feasibility of autonomous, goal-directed systems in applied domains [13,14]. While research on intelligent and agent-based systems predates this period, the integration of learning-based models with planning, memory, and environment interaction relevant to modern agentic AI became substantially more prevalent after 2015. Restricting the review to this period therefore ensured conceptual relevance while avoiding excessive terminological heterogeneity.

### Search strategy and query design

Core search concepts were AI agents and healthcare delivery or clinical care (S1 File). All retrieved records were exported in comma‑separated values or BibTeX formats with standard bibliographic fields including authors, title, year, source, document type, author keywords, abstract, digital object identifier, and author affiliations.

### Data management and deduplication

All retrieved records, exported in BibTeX (PubMed, Web of Science) and CSV (pre-print servers) formats, were initially processed in a unified spreadsheet for metadata standardization, specifically mapping and normalizing fields such as Title, Publication Year, and unique identifiers (DOI/PMID). The entire corpus was then imported into the Covidence environment for management and screening. Deduplication proceeded in two passes. The first pass removed exact matches using standardized digital object identifiers (DOI/PMID). The second pass used Covidence’s fuzzy matching algorithm on normalized title strings combined with first author surnames to identify near duplicates. All candidate duplicates were subjected to independent, duplicate manual review and resolution by two team members to ensure the accurate merging of metadata and to prevent the exclusion of eligible studies due to minor formatting inconsistencies. All subsequent processing steps operated on the final deduplicated corpus of unique studies.

### Selection of sources of evidence

Titles and abstracts were screened independently and in duplicate by the first and last authors against the eligibility criteria after a calibration exercise on an initial subset to harmonize decision rules and refine exclusion rationales. Full texts were obtained for records marked as “include” or “unclear,” and two reviewers independently assessed eligibility at full‑text. Discrepancies were resolved by discussion with adjudication by a third reviewer when consensus could not be reached. Reasons for exclusion at the full‑text stage were recorded in a structured log. The screening process and yields are reported in the results section using a PRISMA flow diagram.

### Data charting

A standardized data charting form was developed and piloted on a subset of eligible studies to ensure clarity and consistency of variable definitions.

### Classification of agent characteristics and evaluation settings

For analytic coherence, agentic systems were classified into conversational agents, workflow or automation assistants, and multimodal decision support agents. Input modality was annotated as text only, speech, vision, or multimodal. Evaluation settings were annotated as simulated environment, laboratory user study, clinical pilot, or real‑world deployment. Outcome classes were mapped to engagement metrics, process measures, diagnostic or prognostic accuracy, clinical outcomes, and safety or usability endpoints. These fields were charted directly from author reports and were used to stratify descriptive statistics and to interpret thematic clusters.

### Critical appraisal

Formal risk of bias assessment was not conducted because the objectives were to map and quantify the scientific landscape rather than to pool effect sizes or to judge comparative efficacy. Methodological features such as randomization, blinding, and allocation concealment were extracted descriptively when reported in trials to contextualize trends in evaluation rigor across publication years and domains.

### Patient and public involvement

There were no patients or members of the public involved in the design, conduct, reporting, or dissemination plans of this study. The work relied exclusively on publicly available bibliographic and published data.

### Ethics and dissemination

The study used publicly available bibliographic records and published materials and did not involve interaction with human participants or access to identifiable private information. Ethical approval was therefore not required. Findings will be disseminated through peer‑reviewed publication and academic presentations, and the indicators are intended to inform research planning and policy discussions, including the equitable development and evaluation of AI agents in settings that have historically been under‑represented in the literature.

### Use of AI tools in manuscript preparation

AI tools were used solely to refine language, syntax, and formatting during manuscript drafting based on human‑generated data, tables, and analysis outputs. No systems were used to perform literature searches, screening, selection, data charting, or quantitative analyses.

## Results

### Study selection

**Fig 1:**
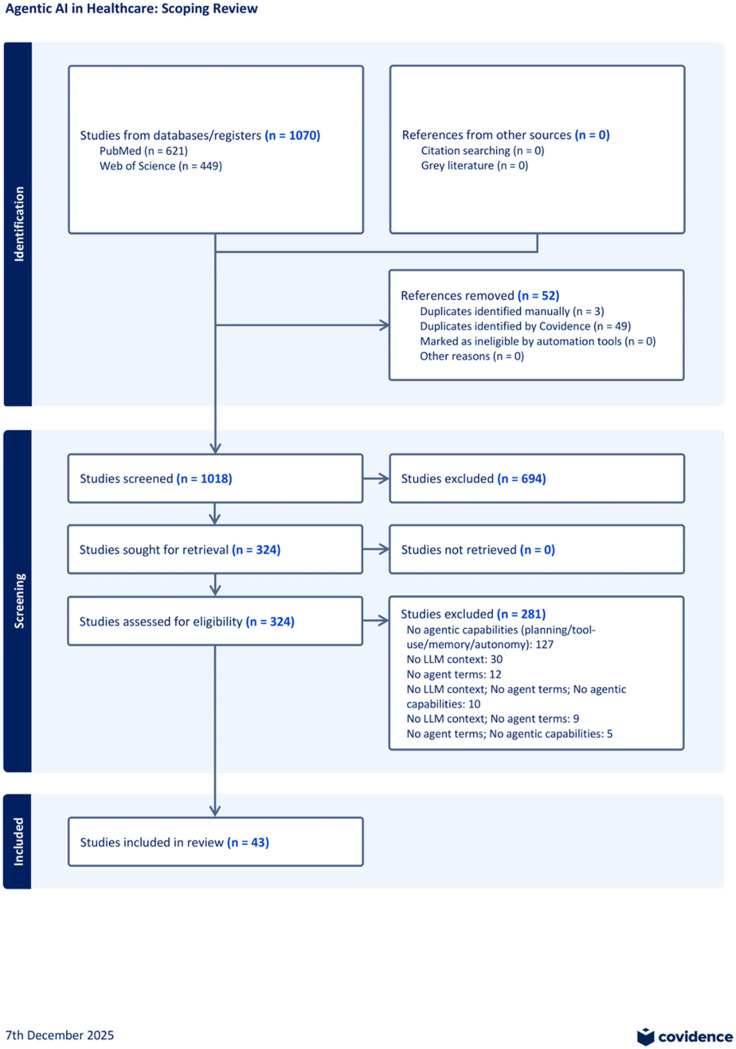
PRISMA Flow Diagram.

The systematic search across all sources yielded 1,070 records. After deduplication, which identified 3 duplicates manually and 49 duplicates via Covidence, a total of 1,018 unique studies were carried forward for title and abstract screening. Of these, 694 studies were deemed irrelevant to the scoping review objectives and were excluded, primarily for not addressing AI or communication concepts. Subsequently, 324 studies were retrieved and assessed at the full-text level for final eligibility. A total of 281 studies were ultimately excluded, with 200 exclusions occurring at the full-text review stage. After the final full-text assessment, 43 studies met all inclusion criteria for the scoping review (Fig 1).

### Summary of included agentic AI systems

The 43 included systems were focused on conversational agents (n = 8), designed for patient interaction or educational purposes, such as virtual patient simulations or mental health support [15,16]; workflow or automation assistants (n = 17), supporting documentation, protocol generation, and data mining tasks, including automating clinical drafting using electronic health record (EHR) data and extracting experimental conditions from bioassays [17,18]; and multimodal decision support agents (n = 18), integrating imaging, structured data, and text for diagnostic or therapeutic planning, exemplified by multi-agent frameworks for medical reasoning and iterative radiotherapy planning optimization [19,20].

Moreover, the included studies detailed the application of agentic AI across various healthcare disciplines, with a concentration in general medicine, biomedical research, and specialized fields including radiology, oncology, and mental health. A substantial majority of the included studies were published in 2025 (36 studies), with the remaining sources split between 2024 (6 studies) and 2026 (2 studies) (Table 1).

**Table 1:** Sources Included in the Agentic AI Scoping Review.

First Author	Publication Year	Healthcare Discipline	Type of AI Agent
Miao, Y. [21]	2026	General Medicine	LLM-based multi-agent
Soman, G. [22]	2025	Mental Health	LLM conversational agent
Saini, A. [23]	2025	Pharmacology	LLM-based multi-agent
Chang, Y. [24]	2024	Radiology	LLM agent with RAG/tool-use
Ngo, H. [25]	2025	Health Informatics	LLM agent with RAG/tool-use
Cammarota, S. [26]	2025	Radiology	LLM planning agent
Zhang, X. [27]	2025	Mental Health	LLM conversational agent
Luo, Y. [28]	2025	Biomedical Research	LLM-based multi-agent
Kumari, M. [29]	2026	General Medicine	LLM-based multi-agent
Wang, Q. [20]	2025	Radiology	LLM-based multi-agent
Chen, H. [30]	2025	General Medicine	LLM agent with RAG/tool-use
Ferber, D. [31]	2025	Oncology	LLM agent with RAG/tool-use
Gorenshtein, A. [32].	2025	General Medicine	LLM-based multi-agent
Gorenshtein, A [33]	2025	General Medicine	LLM-based multi-agent
Kim, H. [17]	2025	General Medicine	LLM agent with RAG/tool-use
Liu, S. [34]	2025	General Medicine	LLM-based multi-agent
Wang, S. [35]	2025	Oncology	LLM agent with RAG/tool-use
Wang, Z. [36]	2025	General Medicine	LLM agent with RAG/tool-use
Zhao, X. [15]	2025	General Medicine	LLM agent with RAG/tool-use
Giske, C. [37]	2024	General Medicine	LLM agent with RAG/tool-use
Imaezue, G. [38]	2025	General Medicine	LLM conversational agent
Zisquit, M. [16]	2025	General Medicine	LLM agent with RAG/tool-use
Altermatt, F. [19]	2025	General Medicine	LLM-based multi-agent
Han, S. [39].	2025	General Medicine	LLM-based multi-agent
Hanna, M. [40]	2025	Pathology	LLM-based multi-agent
Huang, Z. [41]	2025	General Medicine	LLM-based multi-agent
Lin, W. [42]	2025	General Medicine	LLM agent with RAG/tool-use
Lu, M. [43]	2024	Pathology	Clinical copilot
Matsumoto, N. [44]	2025	General Medicine	LLM agent with RAG/tool-use
Ögdü, Ç [45]	2025	General Medicine	LLM-based multi-agent
Pais, C [46]	2024	General Medicine	LLM agent with RAG/tool-use
Mejia, J [47]	2025	General Medicine	LLM-based multi-agent
Niu, Z. [18]	2024	General Medicine	LLM-based multi-agent
Wang, Y. [48].	2025	General Medicine	LLM-based multi-agent
Wang, Y. [49]	2025	General Medicine	LLM-based multi-agent
Yang, E. [50]	2025	Oncology	LLM agent with RAG/tool-use
Li, R. [51]	2025	General Medicine	LLM-based multi-agent
Arowolo, M. [52]	2025	General Medicine	LLM agent with RAG/tool-use
Alghamdi, H. [53]	2024	General Medicine	LLM conversational agent
Yang, E. [54]	2025	Oncology	LLM agent with RAG/tool-use
Vieira-Vieira, C. [55]	2025	General Medicine	LLM-based multi-agent
Yang, E. [56]	2025	Oncology	LLM agent with RAG/tool-use
Deng, L. [57]	2025	General Medicine	LLM-based multi-agent
Chen, X. [58].	2025	General Medicine	LLM-based multi-agent

The core characteristics observed across the included literature were the integration of planning, autonomous tool-use, and explicit self-correction mechanisms, moving beyond simple LLM responses. A significant portion of the evidence described multi-agent systems designed to leverage collaborative reasoning to enhance clinical outcomes. For example, the MedARC framework employed a multi-agent debate mechanism where specialized LLM agents iteratively critiqued and refined medical answers until a consensus goal was met [21]. Similarly, the multi-agent conversation (MAC) framework simulated a multi-disciplinary team discussion for disease diagnosis, with agents evaluating each other’s opinions until consensus was achieved [58]. In clinical workflows, multi-agent systems were developed for complex tasks such as optimizing clinical order sets, using external tools and a feedback mechanism aligned with expert opinion for self-correction [34], or automating complex experimental protocols in biomedical research, as seen in BioResearcher and PrimeGen, which used central controllers to orchestrate specialized agents and engage in self-reflection [28,48]. The BiomedKAI system utilized specialized agents and an Uncertainty-Aware Fallback Routing mechanism that maintained memory of agent accuracy to facilitate a critical self-correcting loop [29].

Single-agent systems demonstrated agency through sophisticated tool utilization and closed-loop execution. Retrieval-augmented generation (RAG) was a near-ubiquitous tool, serving as external memory or a knowledge anchor for applications ranging from oncology research [50] to electrodiagnostic report generation [32]. Several agents demonstrated autonomous code execution: the ESCARGOT agent, designed to generate strategies, executed them via Python code and incorporated a self-debugging loop when errors occurred [44], and a system for creating dengue fever epidemic maps autonomously generated code and implemented iterative correction upon failure [42]. In specialized planning, agents were developed to generate Planning Domain Definition Language (PDDL) files to guide actions for optimal MRI reconstruction [24], and GeneAgent interacted with 18 biological databases via Web APIs for gene-set analysis with a self-verification pipeline [36]. Furthermore, safety and verification were integrated agentically: the multilingual chatbot for Hajj pilgrims incorporated a secondary AI agent to verify medical information against a fact-checking dataset, acting as an evidence-based self-correcting loop to mitigate misinformation [53], while the system for standardized medication directions, MEDIC, utilized deterministic safety guardrails to halt suggestions if parameters were in conflict [46]. In mental health, conversational agents used reinforcement learning and RAG to facilitate continuous learning and self-correction toward the goal of providing empathetic responses [22].

In clinical decision support, LLM agents are designed to mimic complex human analytical processes, such as predicting Alzheimer’s Disease risk by simulating a multidisciplinary team consultation where specialized agents extract symptoms and integrate assessments [51]. Such collaborative systems are also applied to medical question answering, employing agents that iteratively critique and refine responses to enhance factual consistency [21]. A complementary archetype is optimizing resource and technical planning, demonstrated by systems such as GPT-Plan, a multi-agent framework that automates the iterative adjustments necessary for radiotherapy plan optimization, achieving high-quality dosimetric outcomes comparable to experienced human planners [20]. Other planning agents utilize LLM capabilities to translate empirical knowledge into structured actions, such as generating PDDL files to guide optimal parameter tuning for parallel magnetic resonance imaging reconstruction [24]. Furthermore, agents have been developed to accelerate highly specialized research tasks, including extracting experimental conditions from bioassays for pharmacological modeling [18,23] and translating natural language queries into complex terminologies like SNOMED expression constraint language for clinical data analysis [25]. In patient-facing applications, agents serve as conversational partners, applying principles of cognitive behavioral therapy to guide discussions and promote self-reflection in areas such as body image awareness [27] or providing guided support during virtual reality self-talk sessions for psychological counseling [16]. Finally, agents are applied to workflow automation, such as generating assisted electronic medical records and providing bilingual, on-premises clinical drafting within EHR systems [17,30]. The capacity for agency within these systems is conferred through a combination of sophisticated technical mechanisms, with multi-agent frameworks being a central component across clinical and research tasks [51]. These frameworks assign distinct roles to individual LLM-powered agents, such as dosimetrist agent and physicist agent in radiotherapy planning, enabling complex sequential task execution that mirrors human workflow [20].

A core mechanism for conferring up-to-date domain expertise is the integration of RAG, which links the LLM to external data sources. This mechanism functions as the system’s external memory, drawing clinical insights from long-context resources, such as specialized neuromuscular textbooks or biomedical knowledge graphs, to ground interpretations and mitigate hallucinations [23,33,34]. Crucially, agents are equipped with the ability to use external tools to perform necessary actions. These tools include dedicated databases such as OncoKB, RxNav, and EUCAST guidelines, as well as specialized models like MedSAM for image segmentation and Python interpreters for complex calculations, which collectively enhance the agent’s reasoning and strategizing capabilities [31,34,37]. To ensure reliability, agentic systems incorporate explicit self-correction mechanisms. These mechanisms include iterative refinement through structured summarization of agreements and disagreements among agents during debate [21], using deterministic rule-based validation alongside an independent mirror LLM agent to review proposed optimization parameters for logical consistency [20], and utilizing an autonomous self-verification pipeline that compares generated biological claims against curated domain databases [36].

Despite the advanced capabilities demonstrated in laboratory settings, several critical gaps remain concerning the evaluation and real-world clinical integration of LLM-based agentic systems [21]. A key gap exists in clinical efficacy validation, as studies often rely on retrospective clinical data, simulated patient cases, or standardized question-answering benchmarks like PubMedQA and BioASQ rather than prospective trials involving real-world patient outcomes [21,31,51]. Manual evaluations used to assess output quality often suffer from limited sample sizes and the absence of practicing clinicians as evaluators [21]. Furthermore, the long-term impact on clinical workflows is largely unknown, necessitating validation studies to evaluate diagnostic accuracy and resource use in real-world settings [20,31]. Concerns regarding safety and trustworthiness persist, particularly the problem of LLM hallucination, which can compromise output quality despite the use of self-correction loops [20,21,30]. Studies confirm that LLM output often still contains factual errors or inconsistencies, underscoring the need for mandatory human review to ensure patient safety [31,32]. In microbiology diagnostics, the AI agent’s propensity for “over-flagging” suspected resistance mechanisms, despite high sensitivity, raises concerns about generating unnecessary confirmation tests, potentially leading to diagnostic delays and increased costs [37]. Moreover, regulatory bodies currently lack explicit approval for many LLM agents in clinical diagnostics, highlighting the necessity for robust safety and reliability validation before widespread implementation [37].

The integration into existing healthcare workflows presents substantial operational barriers. Systems often struggle with poor usability, complex user interfaces, and the inability to seamlessly integrate with existing EHR systems, leading to low adoption rates among busy clinicians [32,34]. Randomized controlled trials examining AI-assisted interpretation of electrodiagnostic studies found that poor usability scores, particularly regarding efficiency and ease of use, resulted in low physician engagement, despite the agents providing verbose and thorough drafts [33]. Finally, data privacy and security remain major constraints, especially in regions with strict data sovereignty laws. Cloud-based LLMs like GPT-4 are often considered unsuitable for handling sensitive patient data due to regulatory restrictions [17,31], necessitating the exploration of locally deployed, open-source LLMs to mitigate data transfer risks [20].

### Functional archetypes

Agentic systems founded on LLMs are being deployed across three primary functional archetypes in healthcare and biomedical research: clinical decision support and diagnosis, scientific discovery and multi-omics analysis, and operational workflow automation [39,47,48]. In the clinical decision support domain, LLM agents are engineered to mimic human multidisciplinary teams, enhancing the efficiency and reliability of complex decisions [45].

#### Multi-agent coordination and frameworks.

Multi-agent frameworks are used to simulate emergency department staff to perform Korean triage and acuity scale-based classification and treatment planning, demonstrating superior accuracy over single-agent counterparts [39]. Other multi-agent systems specialize in diagnosing rare diseases by engaging doctor agents in interactive conversation until consensus is reached, proving more effective than conventional single-agent LLMs [58]. In scientific discovery and bioinformatics, agents automate complex tasks requiring domain expertise. Conversational agents like AI-HOPE-TGFbeta, AI-HOPE-WNT, AI-HOPE-TP53, and the ARMOA framework are designed for pathway-specific precision oncology, translating natural language queries into executable bioinformatics workflows for colorectal cancer analysis [50,52,54,56]. The BioResearcher system automates the entire dry lab research process, from literature review to experimental protocol generation and code execution [28]. The Alzheimer’s Disease Analysis Model (ADAM-1) uses multi-agent reasoning to integrate microbiome and clinical data for enhanced classification and reporting (Huang, Z., 2025). For operational and logistical automation, systems such as MedScrubCrew orchestrate specialized LLM agents to manage appointment scheduling and patient triage classification based on compatibility matching [47]. The PRINCE multi-agent knowledge engine assists in preclinical drug development by automating complex data retrieval and drafting regulatory documents [55]. Agents also autonomously generate code for public health applications, such as mapping dengue fever epidemics and geographic disasters [42]. The ability of these systems to autonomously plan, reason, and act is rooted in three integrated technical mechanisms: advanced multi-agent orchestration, contextual grounding via retrieval, and iterative self-correction loops. Multi-Agent Coordination and Frameworks. Most complex systems utilize a multi-agent architecture [39,45], often implemented via frameworks such as CrewAI or LangGraph, where specialized LLM instances collaborate [39,47,55,57]. This collaboration often involves a hierarchical structure, such as the use of a Supervisor Agent to orchestrate specialized agents and manage complex user requests [55]. In emergency care simulation, four distinct agents work sequentially to provide coordinated care recommendations [39]. Likewise, for medical case retrieval, a planner agent, a search agent, and a relevance evaluator agent cooperatively refine search queries and assess relevance through iterative interaction [57].

#### Retrieval-augmented generation and tool use.

To overcome inherent knowledge cutoffs and hallucinations in the base LLM, RAG is foundational [29,41,48]. RAG mechanisms often incorporate specialized domain-specific knowledge bases, such as dynamically updated biomedical knowledge graphs [29,44] or custom vector databases indexed with medical textbooks and clinical guidelines [45]. The system BiomedKAI uses a Context-Aware RAG (CARE-RAG) that adapts its retrieval strategy based on the classified intent of the query [29]. Agents actively access external tools to perform tasks beyond simple text generation [45,47,52]. These tools include the RxNorm API for medication management and the DuckDuckGo search engine for web intelligence in triage systems [39,45], database queries (Text-to-SQL) for retrieving structured preclinical data [55], and bioinformatics tools like KEGG and DrugBank for multi-omics analysis [52].

#### Reasoning and self-correction loops.

Agentic reasoning relies on sophisticated planning and self-assessment techniques. Many systems implement Chain-of-Thought (CoT) reasoning to guide step-by-step inference [41,57]. Critically, agents incorporate explicit self-correcting loops to enhance reliability and address errors dynamically [44,45,48]. The adaptive optimizer agent [45] triggers an iterative feedback loop if its diagnostic confidence score falls below a set threshold, refining search parameters and recommending new queries to other agents [45]. In research applications, the Grouped Iterative Validation based Information Extraction (GIVE) method uses continuous extraction and semantic similarity checks (self-reflection) to ensure factual consistency when mining literature [49]. Similarly, the ESCARGOT agent enters a self-debugging loop if Python code generated for execution fails to compile [44].

### Gaps in evaluation and clinical integration

Despite demonstrable advances in performance metrics such as task accuracy and token efficiency in constrained settings [29,45], significant limitations persist regarding the clinical application and evaluation of agentic AI.

#### Long-term clinical efficacy and safety.

A primary challenge is the lack of robust clinical validation in real-world environments [45]. Evaluation frequently relies on idealized, synthetic datasets, such as the Asclepius dataset in triage studies, rather than handling the sparsity, imbalance, and missing values characteristic of real-world patient records [39,41]. Consequently, the actual clinical efficacy of these systems remains largely unconfirmed by prospective studies [45]. Although multi-agent systems aim to reduce hallucination through RAG and self-correction, LLM inconsistency and imperfect reliability persist, necessitating mandatory human oversight for patient safety [41]. Furthermore, ethical concerns arise from the inherent “black box” nature of LLM decision-making, emphasizing the need for improved transparency and explainability in clinical contexts [39]. Systems must also mitigate subtle operational biases, such as an observed tendency for the LLM CDSS to overestimate patient urgency compared to human clinicians [39].

#### Integration into real-world healthcare workflows.

Operational deployment of these systems faces notable barriers. Integrating LLM agents requires seamless interoperability with existing EHR systems, which is currently an ongoing challenge [39,47]. The substantial computational overhead associated with running complex multi-agent architectures and performing real-time retrieval over large document bases results in increased computational cost and latency, potentially limiting practicality in time-critical clinical settings [41,45]. Moreover, the LLM component itself can introduce latency, with single query processing times ranging up to 4.7 seconds, a delay deemed acceptable only for routine, non-emergency clinical uses [45]. Regulatory standards for adaptive AI systems are still developing, creating uncertainty regarding accountability and liability when agents contribute to clinical decisions [40,55]. Many advanced platforms are currently constrained to a research-use-only status, awaiting further validation before they can be generalized to clinical practice [41].

## Discussion

### Summary of findings

The included studies demonstrate that agentic artificial intelligence in healthcare is defined by systems that combine planning, memory, and self-correction to achieve complex clinical and research objectives. For clarity, systems were grouped into conversational agents, workflow or automation assistants, and multimodal decision support agents. Most agents operated on text input, with fewer incorporating speech or vision for multimodal functionality. Evaluation occurred primarily in simulated or laboratory settings, with limited real-world deployment in imaging and pharmacology. Reported outcomes focused on engagement and process efficiency, while diagnostic accuracy was assessed mainly in multimodal systems; clinical outcomes and safety endpoints were rarely addressed. Across categories, conversational agents emphasized usability, workflow assistants targeted operational gains, and multimodal agents concentrated on diagnostic performance. Despite heterogeneity in reporting, the evidence suggests a trend toward integrating planning modules, persistent memory, and external tool use in emerging real-world applications. Multi-agent frameworks were frequently employed to simulate collaborative reasoning, optimize clinical workflows, and enhance decision-making accuracy, while single-agent systems leveraged retrieval-augmented generation and autonomous code execution for specialized tasks.

### Implications and future developments

The emergence of agentic AI systems offers opportunities to enhance diagnostic accuracy, streamline workflows, and support complex decision-making. For clinicians, these systems could reduce cognitive burden and improve efficiency in tasks such as protocol generation, documentation, and multidisciplinary consultations. However, this review highlights persistent gaps in clinical validation, usability, and safety, underscoring the need for rigorous prospective trials and mandatory human oversight to mitigate risks such as hallucinations and operational biases. Health system leaders must prioritize integration strategies that ensure interoperability with existing electronic health record systems, address data privacy concerns, and establish governance frameworks for accountability and regulatory compliance. Without these measures, the promise of agentic AI may remain confined to research settings rather than translating into meaningful improvements in patient care.

In drug discovery, agentic systems are poised to autonomously design, screen, and optimize drug candidates by simulating molecular interactions, accelerating the transition of novel compounds into human trials. Similarly, multi-agent systems will manage intricate clinical trial workflows, from protocol optimization to automated patient recruitment and adaptive randomization, reducing inefficiencies and failure rates. Personalized medicine will benefit from agents capable of integrating multimodal data to deliver highly individualized treatment plans and outcome predictions.

Despite these advances, most current implementations remain text-based and evaluated in laboratory settings, limiting real-world applicability. Future systems will integrate multimodal data streams, including imaging, biosignals, and genomics, enabling comprehensive diagnostic and therapeutic planning. Continuous monitoring agents will analyze real-time data from wearables and hospital systems to detect early signs of deterioration and autonomously escalate interventions. Research will also mature toward patient-specific “digital twins,” computational models that allow simulation of treatment strategies and surgical planning.

Safety, trust, and governance remain paramount. Emerging architectures will incorporate dedicated “Verifier Agents” to audit reasoning and enforce deterministic safety policies before execution. Explainable AI will become essential for transparency and regulatory compliance, while accountability frameworks will define liability for autonomous actions in high-stakes environments.

Finally, seamless integration with electronic health records will be critical for adoption. Future agents will operate ambiently within clinical workflows, autonomously generating documentation and interacting securely with hospital systems through standardized APIs. These developments promise to transform agents from experimental prototypes into invisible, indispensable members of the clinical team.

### Limitations

This scoping review has several limitations. First, the included studies primarily relied on retrospective data, simulated cases, or standardized benchmarks rather than prospective clinical trials, limiting the ability to assess real-world effectiveness. Second, most evaluations were conducted in controlled laboratory settings, which may not reflect the complexity and variability of clinical environments. Third, manual assessments of output quality often involved small sample sizes and lacked input from practicing clinicians, reducing the robustness of performance validation. Fourth, the rapid evolution of agentic AI technologies means that some included systems may already be outdated, and emerging models were not captured within the review timeframe.

## Conclusion

Agentic AI represents a promising evolution in clinical and research applications, distinguished by its capacity for autonomous planning, memory integration, and adaptive tool use. The studies included in this review demonstrate early feasibility across conversational, workflow, and multimodal decision-support systems, yet most evaluations remain confined to simulated or laboratory settings. Evidence of real-world deployment is limited, and outcome reporting rarely extends to patient safety or clinical effectiveness. Future work should prioritize rigorous trials in operational environments, harmonized outcome frameworks, and governance models that ensure transparency and mitigate risk. Advancing these systems from conceptual prototypes to clinically embedded tools will require multidisciplinary collaboration, robust validation, and sustained attention to ethical and regulatory imperatives.

## Supporting information

S1 File Search strategy. Detailed database-specific search strategies used for the identification of studies, including full PubMed, Web of Science, arXiv, and medRxiv queries, controlled vocabulary terms, free-text keywords, Boolean operators, filters, and date limits applied during the literature search.
(DOCX)
