# Supplementary material for: Artificial intelligence agents in healthcare research: A scoping review
Source: PLoS One. 2026 Feb 10;21(2):e0342182. doi: 10.1371/journal.pone.0342182 (PMC12890167; doi:10.1371/journal.pone.0342182)
Supplement: S1 File — Detailed database-specific search strategies used for the identification of studies, including full PubMed, Web of Science, arXiv, and medRxiv queries, controlled vocabulary terms, free-text keywords, Boolean operators, filters, and date limits applied during the literature search. (DOCX) [file pone.0342182.s001.docx]

**Supplemental File 1. Search strategy**

**PubMed**

*1. Agent/System Concepts (AI Agents, Chatbots, etc.)*

| **Terminology Group** | **MeSH Term (Explode for broadest results)** | **Free-Text Keyword (Title/Abstract)** |
| --- | --- | --- |
| Artificial Intelligence | ("Artificial Intelligence"[MeSH]) | ("AI agent"[Title/Abstract] OR "agentic AI"[Title/Abstract]) |
| Conversational Agents | ("Conversational Agent"[MeSH]) | (conversational agent[Title/Abstract] OR chatbot*[Title/Abstract] OR "chat bot"[Title/Abstract] OR "virtual assistant"[Title/Abstract] OR "virtual nurse"[Title/Abstract]) |
| Decision Support | ("Clinical Decision Support Systems"[MeSH]) | (autonomous agent*[Title/Abstract] OR intelligent agent*[Title/Abstract] OR "AI system"[Title/Abstract]) |
| NLP/Speech | ("Natural Language Processing"[MeSH]) | ("virtual coach"[Title/Abstract] OR "dialogue system"[Title/Abstract]) |

*2. Clinical/Context Concepts (Healthcare Delivery, Clinical Care)*

| **Terminology Group** | **MeSH Term (Explode for broadest results)** | **Free-Text Keyword (Title/Abstract)** |
| --- | --- | --- |
| Healthcare/Delivery | ("Delivery of Health Care"[MeSH:NoExp] OR "Health Services Accessibility"[MeSH] OR "Healthcare Facilities, Manpower, and Services"[MeSH]) | (healthcare[Title/Abstract] OR "health care"[Title/Abstract] OR "clinical care"[Title/Abstract] OR medicine[Title/Abstract]) |
| Telemedicine/Informatics | ("Telemedicine"[MeSH] OR "Medical Informatics"[MeSH] OR "Public Health Informatics"[MeSH]) | (telemedicine[Title/Abstract] OR nursing[Title/Abstract] OR "clinical setting"[Title/Abstract]) |
| Core Clinical Topics | ("Diagnosis, Computer-Assisted"[MeSH] OR "Treatment Planning"[MeSH]) | ("clinical decision support"[Title/Abstract] OR "patient engagement"[Title/Abstract] OR "patient education"[Title/Abstract]) |

*3. Combined Query*

Group A: Agent/System Concepts (OR)
("Artificial Intelligence"[MeSH] OR "Conversational Agent"[MeSH] OR "Clinical Decision Support Systems"[MeSH] OR "Natural Language Processing"[MeSH] OR "AI agent"[Title/Abstract] OR "agentic AI"[Title/Abstract] OR conversational agent[Title/Abstract] OR chatbot*[Title/Abstract] OR "chat bot"[Title/Abstract] OR "virtual assistant"[Title/Abstract] OR "virtual nurse"[Title/Abstract] OR autonomous agent*[Title/Abstract] OR intelligent agent*[Title/Abstract] OR "AI system"[Title/Abstract] OR "virtual coach"[Title/Abstract] OR "dialogue system"[Title/Abstract])

Group B: Clinical/Context Concepts (OR)
("Delivery of Health Care"[MeSH] OR "Health Services Accessibility"[MeSH] OR "Healthcare Facilities, Manpower, and Services"[MeSH] OR "Telemedicine"[MeSH] OR "Medical Informatics"[MeSH] OR "Diagnosis, Computer-Assisted"[MeSH] OR "Treatment Planning"[MeSH] OR healthcare[Title/Abstract] OR "health care"[Title/Abstract] OR "clinical care"[Title/Abstract] OR medicine[Title/Abstract] OR telemedicine[Title/Abstract] OR nursing[Title/Abstract] OR "clinical setting"[Title/Abstract] OR "clinical decision support"[Title/Abstract] OR "patient engagement"[Title/Abstract] OR "patient education"[Title/Abstract])

Final Combined Search (AND)
(Group A) AND (Group B)

*4. Filters and Limits*

| **Filter Type** | **Requirement** | **PubMed Format/Action** |
| --- | --- | --- |
| Publication Date | January 1, 2015 to October 31, 2025 | Add date range: 2015/01/01:2025/10/31[pdat] |
| Language | English and French | Limit to English or French in the filter sidebar. |
| Article Type | Higher tiers of evidence, comprehensive syntheses (implicitly targeting full articles/reviews) | Filtering out obvious excluded types (e.g., editorials, letters) and manually reviewing the remaining. The core strategy is broad to enable post-search filtering. |

*5. Full Search String for PubMed*

(((("Artificial Intelligence"[MeSH] OR "Conversational Agent"[MeSH] OR "Clinical Decision Support Systems"[MeSH] OR "Natural Language Processing"[MeSH] OR "AI agent"[Title/Abstract] OR "agentic AI"[Title/Abstract] OR conversational agent[Title/Abstract] OR chatbot*[Title/Abstract] OR "chat bot"[Title/Abstract] OR "virtual assistant"[Title/Abstract] OR "virtual nurse"[Title/Abstract] OR autonomous agent*[Title/Abstract] OR intelligent agent*[Title/Abstract] OR "AI system"[Title/Abstract] OR "virtual coach"[Title/Abstract] OR "dialogue system"[Title/Abstract])) AND (("Delivery of Health Care"[MeSH] OR "Health Services Accessibility"[MeSH] OR "Healthcare Facilities, Manpower, and Services"[MeSH] OR "Telemedicine"[MeSH] OR "Medical Informatics"[MeSH] OR "Diagnosis, Computer-Assisted"[MeSH] OR "Treatment Planning"[MeSH] OR healthcare[Title/Abstract] OR "health care"[Title/Abstract] OR "clinical care"[Title/Abstract] OR medicine[Title/Abstract] OR telemedicine[Title/Abstract] OR nursing[Title/Abstract] OR "clinical setting"[Title/Abstract] OR "clinical decision support"[Title/Abstract] OR "patient engagement"[Title/Abstract] OR "patient education"[Title/Abstract]))) AND 2015/01/01:2025/10/31[pdat])

53,729 results

( ( chatbot*[tiab] OR "conversational agent"[tiab] OR "conversational agents"[tiab] OR "conversational AI"[tiab] OR "dialogue system"[tiab] OR "dialogue systems"[tiab] OR "virtual assistant"[tiab] OR "virtual assistants"[tiab] OR "voice assistant"[tiab] OR "voice assistants"[tiab] OR "AI assistant"[tiab] OR "digital assistant"[tiab] OR "virtual nurse"[tiab] OR "virtual coach"[tiab] OR "health coach"[tiab] OR "autonomous agent"[tiab] OR "autonomous agents"[tiab] OR "intelligent agent"[tiab] OR "intelligent agents"[tiab] OR "AI agent"[tiab] OR "AI agents"[tiab] OR ChatGPT[tiab] OR "GPT-4"[tiab] OR "GPT-3"[tiab] OR "large language model"[tiab] OR "large language models"[tiab] OR LLM[tiab] OR LLMs[tiab] ) AND ( "Delivery of Health Care"[mh] OR "Health Services Accessibility"[mh] OR "Telemedicine"[mh] OR "Medical Informatics"[mh] OR "Diagnosis, Computer-Assisted"[mh] OR "Patient Education as Topic"[mh] OR "Patient Participation"[mh] OR "Nursing"[mh] OR "Patient Care"[mh] OR healthcare[tiab] OR "health care"[tiab] OR clinical[tiab] OR hospital*[tiab] OR "primary care"[tiab] OR outpatient[tiab] OR inpatient[tiab] OR "emergency department"[tiab] OR telehealth[tiab] OR "clinical workflow"[tiab] OR "care delivery"[tiab] OR "patient care"[tiab] ) AND ( clinical[tiab] OR patient*[tiab] OR trial[tiab] OR implement*[tiab] OR deploy*[tiab] OR evaluation[tiab] OR feasibility[tiab] OR effectiveness[tiab] OR "real-world"[tiab] OR "real world"[tiab] ) AND ( "Randomized Controlled Trial"[pt] OR "Controlled Clinical Trial"[pt] OR "Clinical Trial"[pt] OR "Pragmatic Clinical Trial"[pt] OR "Observational Study"[pt] OR "Evaluation Study"[pt] OR "Validation Study"[pt] OR "Comparative Study"[pt] OR "Systematic Review"[pt] OR "Meta-Analysis"[pt] OR (scoping[tiab] AND review[tiab]) OR "umbrella review"[tiab] OR "Guideline"[pt] OR "Practice Guideline"[pt] ) ) NOT ("Case Reports"[pt] OR "Editorial"[pt] OR "Comment"[pt] OR "Letter"[pt] OR "News"[pt]) NOT (animals[mh] NOT humans[mh]) NOT ("Anti-Bacterial Agents"[mh] OR "Antineoplastic Agents"[mh] OR "Anti-Infective Agents"[mh] OR "Pharmaceutical Preparations"[mh]) NOT ("agent-based model"[tiab] OR "agent-based models"[tiab] OR "agent based model"[tiab] OR "agent based models"[tiab]) AND (english[la] OR french[la]) AND ("2015/01/01"[dp] : "2025/10/31"[dp])

1,204 results

**Web of Science**

*1. Agent/System Concepts (AI Agents, Chatbots, etc.)*

| **Concept** | **WoS Search Terms (TS=)** |
| --- | --- |
| Agent/Agentic AI | TS=("AI agent" OR "agentic AI" OR "autonomous agent*" OR "intelligent agent*" OR "AI system*") |
| Conversational Systems | TS=("conversational agent*" OR "virtual assistant*" OR chatbot* OR "chat bot" OR "dialogue system*" OR "virtual coach") |
| Combined Group A | L1 = (Agent/Agentic AI OR Conversational Systems) |

*2. Clinical/Context Concepts (Healthcare Delivery, Clinical Care)*

| **Concept** | **WoS Search Terms (TS=)** |
| --- | --- |
| Healthcare/Clinical | TS=(healthcare OR "health care" OR clinical* OR medicine OR nursing OR telemedicine) |
| Application Contexts | TS=("clinical decision support" OR "patient engagement" OR "patient education") |
| Combined Group B | L2 = (Healthcare/Clinical OR Application Contexts) |

**3. Final Combined Query and Limits**

Group A (L1) - Agent/System Concepts:
TS=("AI agent" OR "agentic AI" OR "autonomous agent*" OR "intelligent agent*" OR "AI system*" OR "conversational agent*" OR chatbot* OR "chat bot" OR "virtual assistant*" OR "virtual nurse" OR "dialogue system*" OR "virtual coach")

Group B (L2) - Clinical/Context Concepts:
TS=(healthcare OR "health care" OR clinical* OR medicine OR nursing OR telemedicine OR "clinical decision support" OR "patient engagement" OR "patient education")

Final Combined Search:
(L1) AND (L2)

**4. Filters and Limits**

| **Filter Type** | **Requirement** | **WoS Action** |
| --- | --- | --- |
| Publication Date | Jan 1, 2015 – Oct 31, 2025 | Time-span filter: Set a Custom Range from 2015-01-01 to 2025-10-31. |
| Language | English and French | Refine Results filter: Select *English* and *French* from the Languages section. |
| Document Type | Interventional/Non-Interventional Studies, Comprehensive Syntheses | Refine Results filter: Focus on relevant types like Article, Review, Proceedings Paper, and Early Access (to capture emergent work), excluding irrelevant types like *Editorial Material* or *Correction*. |

TS=( ( chatbot* OR "conversational agent*" OR "conversational AI" OR "dialogue system*" OR "virtual assistant*" OR "voice assistant*" OR "AI assistant*" OR "digital assistant*" OR "virtual nurse" OR "virtual coach" OR "health coach*" OR "autonomous agent*" OR "intelligent agent*" OR "AI agent*" OR "agentic AI" OR ChatGPT OR "GPT-4" OR "GPT-3" OR "large language model*" OR LLM OR LLMs ) AND ( "health care" OR healthcare OR clinical OR hospital* OR "primary care" OR outpatient OR inpatient OR "emergency department" OR telehealth OR telemedicine OR "clinical workflow" OR "care delivery" OR "patient care" OR nursing OR "patient education" OR "patient participation" OR "delivery of health care" OR "medical informatics" OR "computer-assisted diagnos*" ) AND ( clinical OR patient* OR trial OR implement* OR deploy* OR evaluation OR feasibility OR effectiveness OR "real-world" OR "real world" ) AND ( "randomized controlled trial" OR "randomised controlled trial" OR "controlled clinical trial" OR "clinical trial" OR "pragmatic trial" OR "observational stud*" OR "comparative stud*" OR "evaluation stud*" OR "validation stud*" OR guideline* OR "practice guideline*" ) ) NOT TS=("agent-based model*" OR "agent based model*" OR "agent-based models" OR "agent based models") NOT TS=("anti-bacterial agent*" OR "antibacterial agent*" OR "antineoplastic agent*" OR "anti-infective agent*" OR "pharmaceutical preparation*") NOT TS=("case report" OR "case reports" OR "case report*") AND PY=(2015-2025) AND LA=(English OR French) NOT DT=(Review OR "Review Article" OR "Early Review" OR "Systematic Review" OR "Literature Review" OR "Rapid Review" OR "Review; Book Chapter") NOT DT=("Editorial Material" OR Letter OR "News Item" OR "Book Review" OR Correction OR Retraction OR "Meeting Abstract")

421 results

**arXiv**

*1. Agent/System Concepts (AI Agents, Chatbots, etc.)*

| **Concept** | **arXiv Query String (Term 1)** |
| --- | --- |
| Agent/System | "AI agent" OR "agentic AI" OR "conversational agent" OR chatbot* OR "chat bot" OR "virtual assistant" OR "autonomous agent" OR "intelligent agent" |
| Notes | The search is case-insensitive. Wildcards (*) and phrase searching ("...") are supported. |

*2. Clinical/Context Concepts (Healthcare Delivery, Clinical Care)*

| **Concept** | **arXiv Query String (Term 2)** |
| --- | --- |
| Context/Care | healthcare OR clinical OR medicine OR telemedicine OR nursing OR "clinical decision support" |
| Notes | Terms like clinical are kept broad since the search is field-agnostic (Title/Abstract/All Fields). |

*3. Advanced Search*

- Navigate to the arXiv Advanced Search page.
- Line 1 (Concept A): Enter the Agent/System Query string (using OR operators).
- Line 2 (Concept B): Enter the Clinical/Context Query string (using OR operators).
- The Advanced Search automatically connects these lines with AND, resulting in: (Term 1) AND (Term 2).
- Date Limit: Set the publication date range:
  - From: 2015-01-01
  - To: 2025-10-31
- Language Limit: arXiv does not have a native language filter. Records found must be manually screened for the required English and French language publications.

*4. Full Query*

Term 1 (Agent/System - Line 1):

"AI agent" OR "agentic AI" OR "conversational agent" OR chatbot* OR "chat bot" OR "virtual assistant" OR "autonomous agent" OR "intelligent agent"

Term 2 (Context/Care - Line 2):

healthcare OR clinical OR medicine OR telemedicine OR nursing OR "clinical decision support"

**medRxiv**

*1. Agent/System Concepts (AI Agents, Chatbots, etc.)*

| **Concept** | **medRxiv Query String (Term 1)** |
| --- | --- |
| Agent/System | "AI agent" OR "agentic AI" OR "conversational agent" OR chatbot* OR "chat bot" OR "virtual assistant" OR "autonomous agent" OR "intelligent agent" |
| Notes | Boolean operators (AND, OR, NOT) are supported in the main "Search Terms & Keywords" field. Truncation (*) and phrase searching ("...") are also supported. |

**2. Clinical/Context Concepts (Healthcare Delivery, Clinical Care)**

| **Concept** | **medRxiv Query String (Term 2)** |
| --- | --- |
| Context/Care | healthcare OR clinical OR medicine OR telemedicine OR nursing OR "clinical decision support" |
| Notes | This set of terms ensures the search is specific to health applications. |

**3. Final Combined Query and Limits**

("AI agent" OR "agentic AI" OR "conversational agent" OR chatbot* OR "chat bot" OR "virtual assistant" OR "autonomous agent" OR "intelligent agent") AND (healthcare OR clinical OR medicine OR telemedicine OR nursing OR "clinical decision support")

1. Date Limit:
   - Date Posted (From): Set to 2015-01-01
   - Date Posted (Through): Set to 2025-10-31
   - *This uses the specific date fields available on the medRxiv Advanced Search page.*
2. Language Limit:
   - medRxiv does not offer a native language filter. Records retrieved must be manually screened for the required English and French publications during the screening phase.
